# Comparison between conventional protective mechanical ventilation and high-frequency oscillatory ventilation associated with the prone position

**DOI:** 10.5935/0103-507X.20170067

**Published:** 2017

**Authors:** José Roberto Fioretto, Susiane Oliveira Klefens, Rafaelle Fernandes Pires, Cilmery Suemi Kurokawa, Mario Ferreira Carpi, Rossano César Bonatto, Marcos Aurélio Moraes, Carlos Fernando Ronchi

**Affiliations:** 1 Department of Pediatrics, Faculdade de Medicina de Botucatu, Universidade Estadual Paulista “Júlio de Mesquita Filho” - Botucatu (SP), Brazil.; 2 Faculdade de Educação Física e Fisioterapia, Universidade Federal de Uberlândia - Uberlândia (MG), Brazil.

**Keywords:** Respiration, artificial, Acute lung injury, High-frequency ventilation, Oxidative stress, Acute respiratory distress syndrome, Rabbits, Respiração artificial, Lesão pulmonar aguda, Ventilação de alta frequência, Estresse oxidativo, Síndrome do desconforto respiratório agudo, Coelhos

## Abstract

**Objective:**

To compare the effects of high-frequency oscillatory ventilation and conventional protective mechanical ventilation associated with the prone position on oxygenation, histology and pulmonary oxidative damage in an experimental model of acute lung injury.

**Methods:**

Forty-five rabbits with tracheostomy and vascular access were underwent mechanical ventilation. Acute lung injury was induced by tracheal infusion of warm saline. Three experimental groups were formed: healthy animals + conventional protective mechanical ventilation, supine position (Control Group; n = 15); animals with acute lung injury + conventional protective mechanical ventilation, prone position (CMVG; n = 15); and animals with acute lung injury + high-frequency oscillatory ventilation, prone position (HFOG; n = 15). Ten minutes after the beginning of the specific ventilation of each group, arterial gasometry was collected, with this timepoint being called time zero, after which the animal was placed in prone position and remained in this position for 4 hours. Oxidative stress was evaluated by the total antioxidant performance assay. Pulmonary tissue injury was determined by histopathological score. The level of significance was 5%.

**Results:**

Both groups with acute lung injury showed worsening of oxygenation after induction of injury compared with the Control Group. After 4 hours, there was a significant improvement in oxygenation in the HFOG group compared with CMVG. Analysis of total antioxidant performance in plasma showed greater protection in HFOG. HFOG had a lower histopathological lesion score in lung tissue than CMVG.

**Conclusion:**

High-frequency oscillatory ventilation, associated with prone position, improves oxygenation and attenuates oxidative damage and histopathological lung injury compared with conventional protective mechanical ventilation.

## Abstract (pt)

**Objetivo:**

Comparar os efeitos da ventilação oscilatória de alta frequência e da ventilação mecânica convencional protetora associadas à posição prona quanto à oxigenação, à histologia e ao dano oxidativo pulmonar em modelo experimental de lesão pulmonar aguda.

**Métodos:**

Foram instrumentados com traqueostomia, acessos vasculares e ventilados mecanicamente 45 coelhos. A lesão pulmonar aguda foi induzida por infusão traqueal de salina aquecida. Foram formados três grupos experimentais: animais sadios + ventilação mecânica convencional protetora, em posição supina (Grupo Controle; n = 15); animais com lesão pulmonar aguda + ventilação mecânica convencional protetora, posição prona (GVMC; n = 15); animais com lesão pulmonar aguda + ventilação oscilatória de alta frequência, posição prona (GVAF; n = 15). Após 10 minutos do início da ventilação específica de cada grupo, foi coletada gasometria arterial, sendo este momento denominado tempo zero, após o qual o animal foi colocado em posição prona, permanecendo assim por 4 horas. O estresse oxidativo foi avaliado pelo método de capacidade antioxidante total. A lesão tecidual pulmonar foi determinada por escore histopatológico. O nível de significância adotado foi de 5%.

**Resultados:**

Ambos os grupos com lesão pulmonar aguda apresentaram piora da oxigenação após a indução da lesão comparados ao Grupo Controle. Após 4 horas, houve melhora significante da oxigenação no grupo GVAF comparado ao GVMC. A análise da capacidade antioxidante total no plasma mostrou maior proteção no GVAF. O GVAF apresentou menor escore de lesão histopatológica no tecido pulmonar que o GVMC.

**Conclusão:**

A ventilação oscilatória de alta frequência, associada à posição prona, melhora a oxigenação, e atenua o dano oxidativo e a lesão pulmonar histopatológica, comparada com ventilação mecânica convencional protetora.

## INTRODUCTION

Mechanical ventilation (MV) is the most important treatment for acute respiratory distress syndrome (ARDS) and is capable of modifying the evolution of the disease.^(1)^ Although protective conventional MV (CMV) is effective in many patients, a significant number present with severe respiratory failure, in which CMV may not guarantee oxygenation and ventilation. In these cases, when pulmonary protection is required, high-frequency oscillatory ventilation (HFOV) becomes an interesting therapeutic alternative^(2)^ because it uses a tidal volume (TV) lower than the anatomical dead space volume and frequency higher than the physiological one, avoiding elevated pressures and alveolar volumes typical of CMV.^(3-5)^

Due to the high mortality observed in ARDS, additional therapeutic strategies for MV have been developed, especially for the prone position.^(6)^ In ARDS, lung injury is heterogeneous and varies with the position of the patient, being more significant in areas that depend on gravity, i.e., the dorsal lung region, when the patient is in the supine position.^(7,8)^ The prone position may improve gas exchange by redistributing ventilation to better-perfused dorsal lung areas^(9,10)^ and by mediating homogenization of TV distribution associated with changes in chest wall mechanics,^(11)^ alveolar recruitment,^(12)^ and redirection of compressive forces exerted by the weight of the heart on the lungs,^(13)^ resulting in better removal of secretions. Recently, studies have shown that there is improved survival in patients treated early with prone position.^(14)^

Considering the protective characteristics of HFOV and its capacity to redistribute ventilation to better-perfused lung areas, which results in better oxygenation in ARDS, and the potential recruitment of prone position, our hypothesis is that the sum of the beneficial effects of HFOV and prone position improves oxygenation more, makes histopathological lesions more homogeneous and of lower intensity, and attenuates oxidative damage to pulmonary tissue when compared with CMV associated with prone position.

The present study aimed to compare the effects of prone position associated with HFOV and CMV by oxygenation, histology, and pulmonary oxidative damage in an experimental model of acute lung injury induced in rabbits.

## METHODS

This study was conducted at the Experimental Laboratory of the Center for Clinical and Experimental Research of the Department of Pediatrics of the *Faculdade de Medicina de Botucatu* of the *Universidade Estadual Paulista "Júlio de Mesquita Filho"* (UNESP) and was approved by the Ethics Committee on Animal Experimentation of the *Faculdade de Medicina de Botucatu* under protocol number 795.

A prospective study *in vivo* conducted on laboratory animals. White male rabbits provided by the School of Medicine Vivarium - Botucatu *Campus* were used, weighing 2.0 to 3.0kg.

The instrumentation of the animals followed a protocol already established by the group.^(15,16)^ Briefly, after being weighed, the animals were anesthetized and sedated with a solution of ketamine (50mg/kg) and acepromazine (2mg/kg) administered intramuscularly. Animals were placed in a surgical brace, received 100% oxygen through a nasal catheter, and underwent cervical and thoracic trichotomy for the placement of heart rate (HR)-monitoring electrodes. If the HR decreased to below 180bpm, atropine was given at a dose of 0.01mg/kg intravenously in the auricular vein. The anterior region of the animal's neck was anesthetized with xylocaine to perform the tracheostomy. A tracheal tube of the highest possible caliber (3.0 to 3.5mm internal diameter, Portex, Hythe, UK) was inserted through the tracheostomy and was held in position with surgical tape. MV was then immediately started with the CMV apparatus (Inter^®^ 7 plus, Oxy System, São Paulo (SP), Brazil). The initial parameters were as follows: pressure-regulated volume-controlled mode, with a target TV of 6mL/kg; respiratory rate (RR) of 40 cycles per minute, adjusted according to the partial pressure of carbon dioxide (PaCO_2_); inspiratory time (Ti) of 0.5 second; positive end-expiratory pressure (PEEP) of 5cmH_2_O; and inspired oxygen fraction (FiO_2_) of 1.0. These parameters were maintained for a stabilization period of 10 minutes, until the moment of lung injury induction in the treated groups. After the tracheostomy, the carotid artery and the internal jugular vein were dissected. A single-lumen vascular catheter was inserted into the common carotid (22 Gauge Jelco, Introcan^®^ Safety^TM^, B-Braun, Melsungen, Germany), and a double-lumen catheter (5Fr, Arrow International Inc., Reading, Philadelphia, USA) was inserted in the superior vena cava through the jugular vein. The arterial catheter was used to obtain blood gases and for continuous monitoring of mean arterial pressure (MAP) using a pressure monitoring system (LogicCal^®^ from Medex, Dublin, USA) connected to a multiparameter monitor (Dixtal, Manaus, Brazil). The vena cava catheter was used for administration of continuous infusion sedatives, maintenance fluids, and vasoactive drugs.

Once the vascular accesses were obtained, anesthesia was maintained by continuous intravenous administration of 10mg/kg/hour of ketamine until the conclusion of the experiment. In addition, the animals were submitted to neuromuscular blockade by intravenous administration of 0.2mg/kg pancuronium, and the blockade was maintained with additional doses of 0.1mg/kg as required to control respiratory movements. At any time in the experiment, if MAP reached values below 50mmHg, continuous intravenous infusion of noradrenaline was initiated at an initial dose of 0.2µg/kg/minute; if there was no response, the dose was gradually increased to 1µg/kg/minute. The body temperature was monitored using a digital rectal thermometer and was maintained between 38°C and 40°C using heat packs, and the blood volume was maintained by continuous infusion of 4mL/kg/hour of saline solution plus 5% dextrose.

### Induction of the acute lung injury model

Acute lung injury (ALI) was induced according to a previously described technique.^(15,17-19)^ Briefly, six successive washes of the lung were performed with warm saline (38°C) in aliquots of 30mL/kg, at a maximum pressure of 30cmH_2_O, through the tracheal cannula. Each washing procedure lasted 60 seconds, 20 seconds being reserved for infusion and the remaining time for withdrawal, which was performed by gravity and external chest compression movements. After completion of the withdrawal, the procedure was repeated every 3 - 5 minutes until reaching a PaO_2_/FiO_2_ < 100mmHg, which was confirmed after 10 minutes of stabilization. If the criterion was not reached, two more washes were performed in the sequence and, after 10 minutes, a new gasometry was obtained, and so on, until a PaO_2_/FiO_2_ < 100mmHg was reached. After satisfying this criterion, the animals were randomized to create the experimental groups.

### Experimental groups and mechanical ventilation parameters

**Figure 1:**
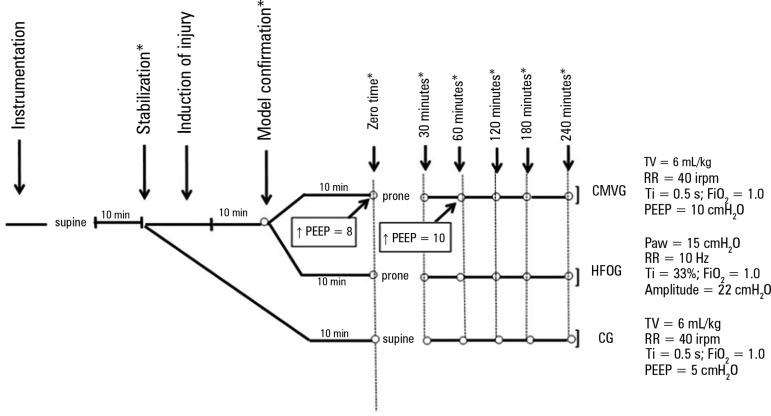
Experimental protocol and distribution of animals according to the type of ventilation used. CMVG - conventional mechanical ventilation group; TV - tidal volume; RR - respiratory rate; Ti - inspiratory time; FiO_2_ - inspired oxygen fraction; PEEP - positive end-expiratory pressure; Paw - mean airway pressure; HFOG - high-frequency oscillatory ventilation group; CG - control group. * Gasometry collection.

Based on previous studies performed with similar methodologies, the animals were distributed in three groups of 15 rabbits each, as follows: instrumented healthy animals (control - CG), maintained in supine position and submitted to CMV in pressure-regulated volume-controlled mode, with TV of 6mL/kg, RR of 40 cycles per minute, a Ti of 0.5 seconds, a PEEP of 5cmH_2_O and an FiO_2_ of 1.0; animals with ALI submitted to protective CMV (conventional mechanical ventilation group - CMVG) in prone position, with the same initial parameters described for CG. In this group, PEEP was increased to 8cmH_2_O during the first hour and then to 10cmH_2_O, and then was maintained until the end of the experiment. Animals with ALI underwent HFOV in prone position with a mean airway pressure of 15cmH_2_O, an RR of 10Hz, a Ti of 33%, a pressure range of 22cmH_2_O, and an FiO_2_ of 1.0, in the mechanical ventilator SensorMedics 3100A (Viasys Healthcare, Yorba Linda, USA), with RR and amplitude adjusted to maintain PaCO_2_ at physiological levels (35 - 45mmHg), forming the high-frequency oscillatory ventilation (HFOG) group (Figure 1).

Ten minutes after the beginning of the specific ventilation of each group, new gasometry was obtained, with this timepoint being called time zero (T0), after which the animals were placed in prone position. From this moment, they were ventilated for 4 hours, and arterial blood gas measurements were collected at moments 30, 60, 120, 180, and 240 minutes. The time of 4 hours was chosen, taking into account the viability of the rabbits in this type of experiment, based on previous experiments and the studies cited above, which demonstrated early clinical and experimental effects of the prone position.^(17,18,20)^

### Manipulation of the lungs and determination of tissue injury. Pulmonary histology

At the end of the experiment, the animals received 1 mL of heparin and then underwent euthanasia by rapid intravenous administration of ketamine. Subsequently, the tracheal tube was occluded, and the thorax opened to exclude the presence of occult pneumothorax, to confirm the position of the vascular catheters and tracheal tube, and to collect samples for histological analysis and bronchoalveolar lavage. In animals in which bronchoalveolar lavage was performed (n = 8), the right bronchus was ligated by surgical tape, the lung/heart block was removed, the left lung was washed twice using aliquots of 15mL/kg of normal saline, and the drained fluid was collected for analysis. In the animals submitted to histological analysis (n = 7), the trachea/lung/heart block was removed, the lungs and trachea were separated from the heart, and the left lung of animals not submitted to bronchoalveolar lavage was filled with 10% formalin solution. Filling was achieved by means of a column with serum equipment 30cm long, with a vial containing formalin connected to one of its ends and the trachea of the animal connected to the other end. From this system, the formaldehyde slowly dripped by gravity to fill the alveolar spaces, preserving their architecture. After a minimum of 24 hours of fixation, fragments were embedded in paraffin, and axial sections of the lung were then stained with hematoxylin and eosin and examined by two pathologists in a blind and independent manner. In each slide, the specimen was divided into two distinct zones, representing the dependent (dorsal) and non-dependent (ventral) regions of the lung. Ten microscopic fields were randomly selected for the examination, five in each region, totaling 50 analyses for each animal. Pulmonary histological lesions were quantified by a score composed of seven variables (alveolar and interstitial inflammation, alveolar and interstitial hemorrhage, edema, atelectasis, and necrosis). The severity of the lesion was classified for each of the seven variables as follows: zero if no lesion was observed; 1 if injured in 25% of the field; 2 if injured in 50% of the field; 3 if injured in 75% of the field; and 4 if diffuse injury. The maximum possible score was 28, and the minimum score was zero.^(21,22)^

### Concentration of malondialdehyde

Concentrations of malondialdehyde (MDA), a marker of lipid oxidative damage, were measured in pulmonary lavage fluid and plasma using the method of Esterbauer et al.^(23)^

### Pulmonary oxidative stress: total antioxidant performance assay

Lung oxidative stress was evaluated using the total antioxidant performance (TAP) assay described by Aldini et al.^(24)^ Briefly, TAP assay, validated by Beretta et al.,^(25)^ determines the antioxidant capacity by measuring oxidative stress and is the only approach that captures the antioxidant network of the lipophilic and hydrophilic compartments and their interactions.^(26)^ It is based on the generation of lipophilic radical (MeO-AMVN) and an oxidizable lipophilic substrate (BODIPY), which specifically measures the oxidation of the lipid compartment related to the actions of liposoluble and water-soluble antioxidants through a mechanism of synergism and cooperation.^(27)^

For each sample, 100µL of plasma and 100µL of phosphatidylcholine (PC) standard (PC1 and PC2) were pipetted separately. In plasma and in both PCs, 300µL of ice-cold phosphate-buffered saline (PBS) (pH 7.4) was added, and 100µL of BODIPY was then added to all samples; after a water-bath, 420µL of PBS and 80µL of 2,2' - azobis (2-amidinopropane) dihydrochloride (AAPH) were pipetted into each sample. Samples were vortexed and then placed on a plate for analysis using the Wallack Victor X2 apparatus (Perkin-Elmer, Boston, USA) and the WorkOut 2.5 program (Dazdaq Solutions Ltd.). The entire procedure was performed under indirect light, and the samples were prepared in triplicate.

### Statistical analysis

Variables with normal distribution were compared among the different experimental groups using analysis of variance (ANOVA), with subsequent multiple comparisons between pairs using the Bonferroni test. Variables with non-normal distribution were compared among the different groups using Kruskal-Wallis ANOVA, with subsequent comparisons by Dunn's test. The analysis of the behavior of a variable over time, in cases of normal distribution, was evaluated using repeated measures ANOVA, with comparisons between pairs using Bonferroni's test; in cases of non-normal distribution, Friedman's test for repeated measures was used, with later comparisons by Dunn's method. A *t-*test was used to compare the number of lung washes between the two treated groups. Statistical significance was defined as p < 0.05.

## RESULTS

### Hemodynamics, pulmonary mechanics, and gas exchange

There were no significant differences between groups regarding animals weight and number of washes required for lesion induction. Likewise, there were no significant differences among groups regarding PaO_2_/FiO_2_ ratio, oxygenation index (OI), lung compliance, and MAP compared moments before and after lung injury induction. Comparison before and after lung injury within each group indicated that there was a significant worsening of oxygenation and a decrease in pulmonary compliance in both groups after induction, as shown in table 1.

**Table 1:** Comparison of experimental groups in relation to partial pressure of oxygen/inspired oxygen fraction, oxygenation index, pulmonary compliance, and mean arterial pressure, before and after injury

Variables	CG N = 15	HFOG N = 15		CMVG N = 15	
	Baseline values	Before the LI	After the LI	Before the LI	After the LI
PaO_2_/FiO_2_	444.26 ± 59.45	445.31 ± 54.17	70.23 ± 21.35*	465.86 ± 48.30	68.93 ± 11.60*
Oxygenation index (cmH_2_O/mmHg)	1.95 ± 0.32	2.25 ± 0.80	20.10 ± 12.92*	1.95 ± 0.46	14.23 ± 2.28*
Compliance (mL/cmH_2_O)	1.73 ± 0.62	2.06 ± 0.49	0.81 ± 0.19*	1.76 ± 0.41	0.74 ± 0.25*
MAP (mmHg)	60.13 ± 15.61	61.89 ± 14.47	69.55 ± 9.45*	65.2 ± 15.84	68.5 ± 15.05*

CG - control group; CMVG - conventional mechanical ventilation group; HFOG - high-frequency oscillatory ventilation group; LI - lung injury; PaO_2_ - partial pressure of oxygen; FiO_2_ - inspired oxygen fraction; MAP - mean arterial pressure.

* p < 0.05 comparing the moments before and after the induction within each group. Normal distribution: *t* test. Non-normal distribution: Mann-Whitney rank.

In the evaluation of the hemodynamic state, MAP was not significantly different between the moments during the experiment, indicating homogenization of the groups and strict control of the variable, using, with vasoactive drugs administered when necessary. The percentages of animals requiring vasoactive drug were 20% in CG and 26% in HFOG and CMVG.

**Figure 2:**
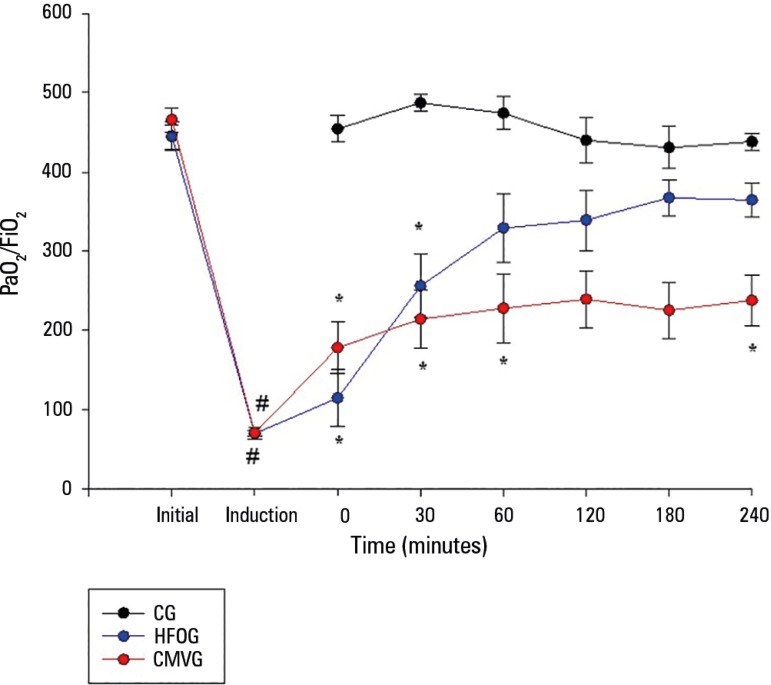
Evolution of oxygen partial pressure/inspired oxygen fraction in the experimental period (up to 240 minutes). PaO_2_ - partial pressure of oxygen; FiO_2_ - inspired oxygen fraction; CG - control group; HFOG - high-frequency oscillatory ventilation group; CMVG - conventional mechanical ventilation group. * p < 0.05 for the high-frequency oscillatory ventilation and conventional mechanical ventilation groups compared with the control group; # p < 0.05 in relation to the initial moment.

After lesion induction, the groups developed significant hypoxemia compared to the beginning of the experiment. After 4 hours of CMV, the HFOG showed a significant improvement in oxygenation compared with the CMVG, presenting a PaO_2_/FiO_2_ ratio similar to the moments before injury induction and to the CG, as shown in figure 2.

### Oxidative stress - malondialdehyde and total antioxidant performance

**Figure 3:**
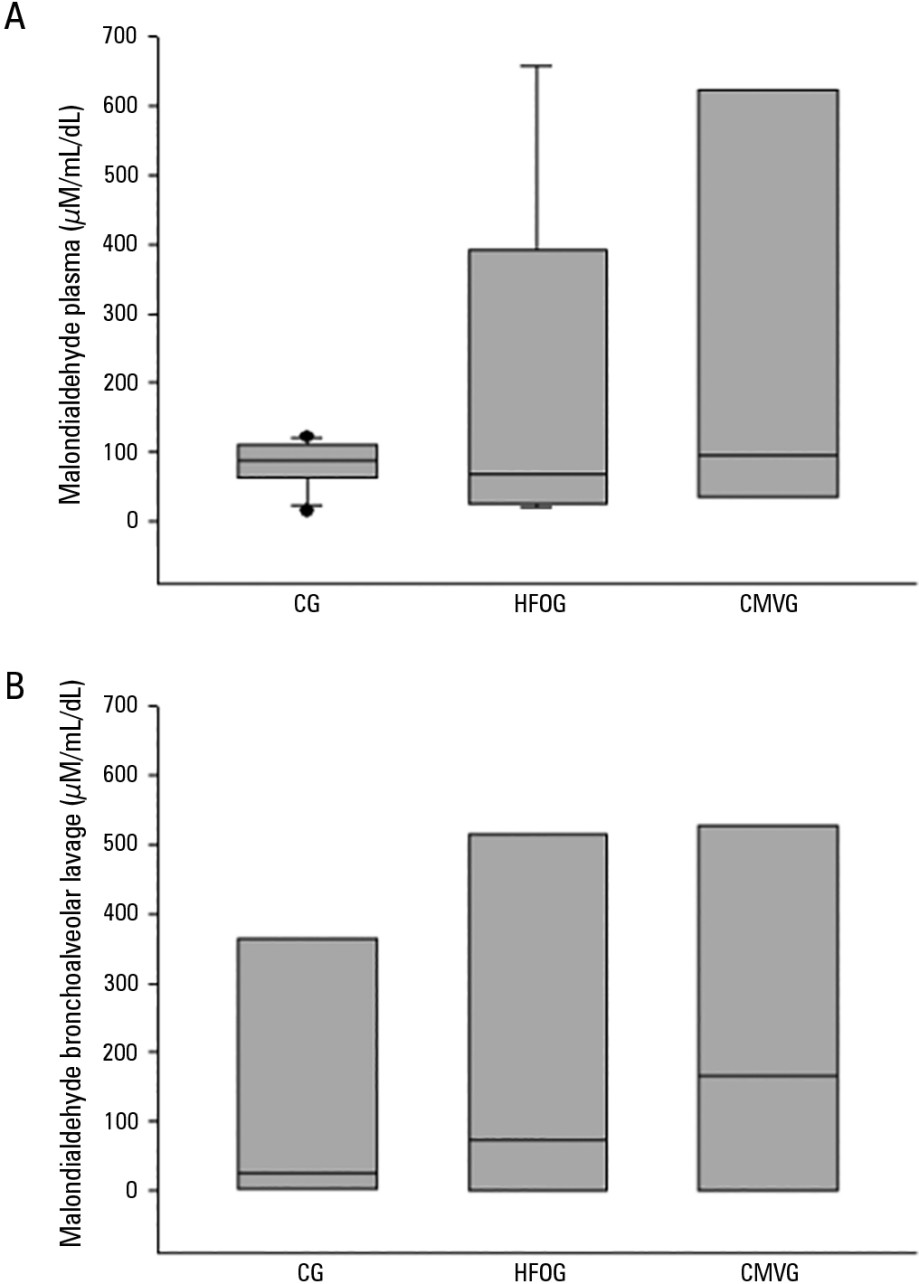
Concentrations of malondialdehyde in each group: (A) Plasma: High-frequency oscillatory ventilation group [control group: 87.38 (64.20 - 106.34) > high-frequency oscillatory ventilation group: 67.63 (26.40 - 327.60) < conventional mechanical ventilation group: 95.92 (34.49 - 599.06); p < 0.05). (B) Bronchoalveolar lavage: [control group: 25.75 (2.74 - 291.86) < high-frequency oscillatory ventilation group: 72.63 (0.75 - 449.64) < conventional mechanical ventilation group: 167.15 (1.85 - 462.20); p > 0.05]. CG - control group; HFOG - high-frequency oscillatory ventilation group; CMVG - conventional mechanical ventilation group. The bars above and below the rectangles indicate the 25^th^ and 75^th^ percentiles, and the inner bar indicates the median.

There were no significant differences between the groups when MDA levels were evaluated in plasma and bronchoalveolar lavage (Figure 3).

**Figure 4:**
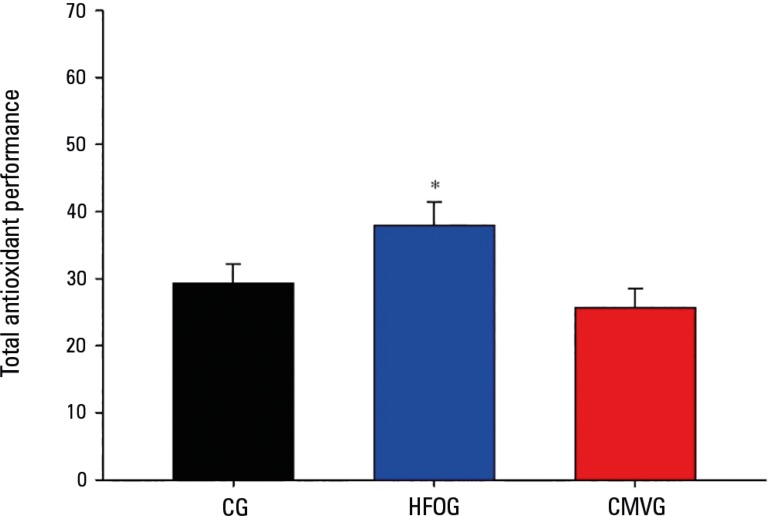
Total antioxidant performance in plasma for each group. CG - control group; HFOG - high-frequency oscillatory ventilation group; CMVG - conventional mechanical ventilation group. * p < 0.05.

Regarding the evaluation of TAP in plasma, HFOG presented similar antioxidant protection to CG and significantly higher protection than CMVG, as shown in figure 4.

### Histopathology

**Figure 5:**
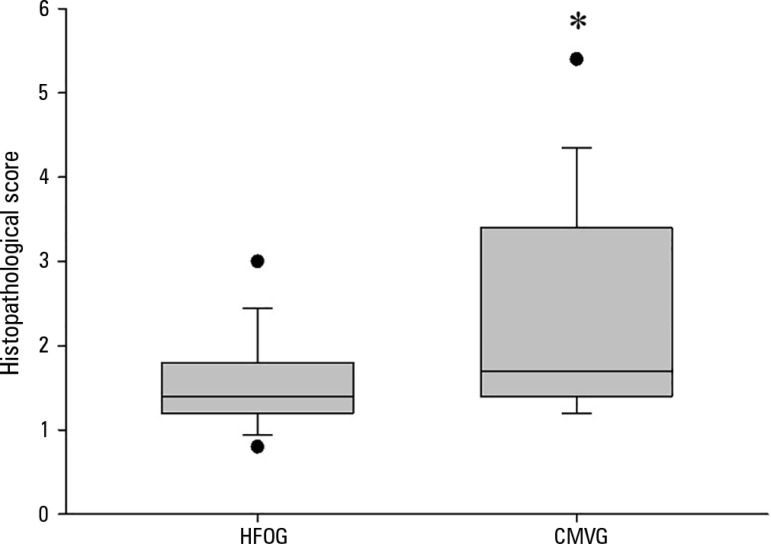
Histopathological lesion score in lung tissue (high-frequency oscillatory ventilation group: 1.4 (1.2 - 1.8) < conventional mechanical ventilation group: 1.7 (1.4 - 3.2); * p < 0.05]. The lower edges of the rectangles indicate the 25^th^ percentiles, the horizontal lines within the rectangles mark the medians, and the upper edges indicate the 75^th^ percentiles. The bars above and below the rectangles indicate the percentiles 90 and 10, respectively, and the filled circles represent individual values. HFOG - high-frequency oscillatory ventilation group; CMVG - conventional mechanical ventilation group.

HFOG presented a significantly lower histopathological lesion score than did CMVG, as shown in figure 5.

## DISCUSSION

Recently, our group was the first to publish the results of a comparison between protective CMV and HFOV regarding total antioxidant performance by TAP assay and concluded that HFOV attenuated oxidative stress.^(15)^

Few studies have evaluated the association of HFOV with prone position.^(28,29)^ Clinical studies have concluded that prone position associated with CMV or HFOV improves oxygenation in 12 hours, in contrast to the supine position associated with HFOV, in addition to decreasing pulmonary inflammation. Demory et al.^(29)^ suggested that HFOV is able to maintain prone position-induced alveolar recruitment, and its use after the prone position allows for the reduction of FiO_2_ to potentially less toxic levels.

In the present study, 4 hours after initiation of the experiment, HFOG showed a significant improvement in oxygenation, presenting values similar to those prior to lesion induction, corroborating an earlier study by our group,^(15)^ also performed in rabbits with ALI induced by infusion of saline in animals ventilated with HFOV in supine position. This finding confirms our hypothesis that in cases of severe hypoxemia, HFOV may be an attractive alternative for more effective oxygenation improvement.^(2)^

Regarding oxidative stress, the plasma MDA concentration was lower in HFOG than in CMVG but did not reach statistical significance. However, when oxidative stress was evaluated by TAP, there was greater pulmonary protection in HFOG compared with CMVG animals. This result may have been due to the evaluation characteristics of the TAP assay, which is more sensitive when measuring the TAP of the two compartments (hydrophilic and lipid) present in the biological samples.^(24)^ Still, this result shows that there was greater pulmonary antioxidant protection in the HFOG compared with that in the CG animals. We believe that this behavior of HFOG in relation to TAP occurred since CMV alone can damage the healthy lung by the cyclical opening and closing movements of alveolar units, whereas HFOV provides greater lung protection by maintaining a constant lung volume.^(27)^ This result is in agreement with the findings of Ronchi et al.,^(15)^ who also used this method and obtained values similar to those in the CG in the group ventilated with HFOV and significantly higher than those in the CMV group insupine position. Reinforcing our findings, in a study conducted by Mazullo Filho et al.,^(30)^ the authors evaluated 12 patients admitted to the intensive care unit, comparing the first and last days of use of CMV, and observed that patients had increased markers of oxidative stress and reduced antioxidant enzyme levels due to the use of CMV.

Histopathological findings typical of ARDS in this model include edema, polymorphonuclear infiltrate in the alveolar space, hyaline membrane formation, and capillary congestion,^(21)^ which were evaluated by histological scores, including inflammation, hemorrhage, edema, atelectasis, and necrosis.^(22,31)^ We have demonstrated that the HFOG presented significant reductions in histopathological lesions when compared with CMVG. Corroborating our findings, an experimental study in pigs,^(31)^ in which ARDS was induced by lavage with saline, showed that HFOV associated with prone position led to a reduction in the histopathological score when compared with CMV animals. In addition, there was an improvement in oxygenation, a significant reduction in pulmonary *shunt* fraction, and normalization of cardiac output with lower mean airway pressures when HFOV was associated with supine position.

The present study has some limitations. First, there is no animal model capable of reproducing all of the characteristics of ALI/ARDS in humans. However, one of the most widely used ALI models in animals is alveolar lavage with heated saline, which causes surfactant depletion, resulting in lung injury very similar to that of ARDS in humans. In addition, the 4-hour experiment under FiO_2_ of 1.0 may lead to lung parenchymal damage and can interfere with the oxidative metabolism of these animals. In contrast, the use of the same oxygen concentration and the definition of ventilatory parameters for all groups likely excluded any significant variations among groups due to oxygen toxicity. The choice of the number of animals was based on previous studies, and no sample calculations were performed.

## CONCLUSION

High-frequency oscillatory ventilation in association with prone position improves oxygenation and leads to reduced oxidative damage, as measured by total antioxidant performance assay and attenuation of histopathological lung injury, compared with protective conventional mechanical ventilation in prone position.
